# HIV Capsid and Integration Targeting

**DOI:** 10.3390/v13010125

**Published:** 2021-01-18

**Authors:** Alan N. Engelman

**Affiliations:** 1 Department of Cancer Immunology and Virology, Dana-Farber Cancer Institute, Boston, MA 02215, USA; alan_engelman@dfci.harvard.edu; Tel.: +1-617-632-4361; 2 Department of Medicine, Harvard Medical School, Boston, MA 02115, USA

**Keywords:** HIV, capsid, nuclear import, integration, integration targeting, CPSF6, antiviral inhibitor

## Abstract

Integration of retroviral reverse transcripts into the chromosomes of the cells that they infect is required for efficient viral gene expression and the inheritance of viral genomes to daughter cells. Before integration can occur, retroviral reverse transcription complexes (RTCs) must access the nuclear environment where the chromosomes reside. Retroviral integration is non-random, with different types of virus-host interactions impacting where in the host chromatin integration takes place. Lentiviruses such as HIV efficiently infect interphase cells because their RTCs have evolved to usurp cellular nuclear import transport mechanisms, and research over the past decade has revealed specific interactions between the HIV capsid protein and nucleoporin (Nup) proteins such as Nup358 and Nup153. The interaction of HIV capsid with cleavage and polyadenylation specificity factor 6 (CPSF6), which is a component of the cellular cleavage and polyadenylation complex, helps to dictate nuclear import as well as post-nuclear RTC invasion. In the absence of the capsid-CPSF6 interaction, RTCs are precluded from reaching nuclear speckles and gene-rich regions of chromatin known as speckle-associated domains, and instead mis-target lamina-associated domains out at the nuclear periphery. Highlighting this area of research, small molecules that inhibit capsid-host interactions important for integration site targeting are highly potent antiviral compounds.

## 1. Introduction

**Figure 1:**
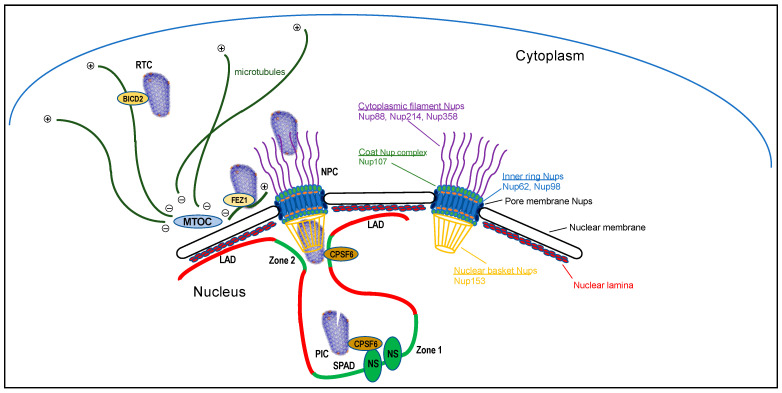
Intracellular HIV-1 trafficking to active genes for integration (not drawn to scale). The RTC, modeled after protein data bank (PDB) code 3J3Y (see Figure 2D), is shown interacting with a microtubule for retrograde transport via CA-binding protein BICD2. After reaching the microtubule organizing complex (MTOC), antegrade transport via kinesin-FEZ1 may shuttle the RTC to the NPC for nuclear transport. Nup proteins that have been shown to interact with CA are highlighted to the right, color coded to demarcate their relative positions within the NPC and noted subcomplexes. During nuclear entry, CPSF6 aids the release of the RTC from Nup153/the NPC nuclear basket [17]. Following nuclear entry, the preintegration complex (PIC) traffics to nuclear speckles (NSs) for integration into speckle-associated domains (SPADs) in a manner that is dependent on the CA-CPSF6 interaction [12]. Lacking this interaction, PICs mislocalize to the nuclear periphery and uncharacteristically target lamina-associated domains (LADs) for integration [15,18]. The PIC is depicted partially uncoated. Transcriptionally active Zone 1 and Zone 2 regions of chromatin are highlighted by green color [13].

Retroviruses are the only type of animal virus that routinely recombine their genetic information with that of the infected host organism. Retroviruses have accordingly evolved to harbor their own specialized DNA recombination enzyme, which is called integrase (IN). The nucleoprotein complex within the HIV-1 virion particle consists of two copies of plus-stranded genomic RNA bound by the viral nucleocapsid (NC) protein and reverse transcriptase (RT) and IN enzymes. The virion particle undergoes a series of transitional changes as it moves from the extracellular environment through the cell to reach the chromosomal DNA targets of integration within the nucleus. Cellular entry via membrane fusion delipidates the virus particle and releases the viral core into the cytoplasm. RT converts the two copies of HIV-1 RNA into one double-stranded DNA molecule within the confines of the reverse transcription complex (RTC), which in essence is the reverse transcription-competent viral core [1,2,3] (Figure 1). IN enzyme activity ensues within the confines of the preintegration complex (PIC), wherein a multimer of IN binds and bridges the two ends of the viral DNA together [4,5,6] to form the intasome nucleoprotein complex (reviewed in [7]). Following integration, the provirus in most cases serves as an efficient transcriptional template that is stably inherited to daughter cells upon cell division. On rare occasion, the provirus enters transcriptional latency. Reactivation of latent proviruses following the cessation of antiretroviral therapy is the main barrier precluding HIV cure [8].

Retroviral reverse transcripts are integrated into host cell genomes in non-random fashions (reviewed in [9]). HIV-1 integration favors transcriptionally active genes and chromatin [10,11] including regions known as speckle-associated domains (SPADs) [12] due to their physical proximity to nuclear speckles [13]. HIV-1 integration at the same time disfavors heterochromatin including lamina-associated domains (LADs) [14,15] that are in close contact with the proteinaceous nuclear lamina at the peripheral regions of the nucleus [16] (Figure 1).

Research over the past 15 years has highlighted roles for specific virus-host interactions in retroviral integration targeting (see [19] for a recent review). Due to its central role in mediating integration, it is unsurprising that IN-binding cellular factors play prominent roles in integration targeting, and the lentiviral IN binding protein lens epithelium-derived growth factor (LEDGF)/p75 plays a key role in the genic integration targeting profile of HIV-1. Reduction of cellular LEDGF/p75 protein levels via mRNA-directed knockdown [20] or stable knockout of the *PSIP1* gene that encodes for LEDGF/p75 [21,22,23] significantly reduced the frequency of intragenic HIV-1 integration targeting. Moreover, the genic profiles of the residual proviruses that formed under these conditions uncharacteristically congregated toward the upstream regions of genes [24,25,26]. Consistent with these observations, LEDGF/p75 has been shown to interact with mRNA splicing factors [24,27] and can overcome the transcriptional block imposed by nucleosomes in vitro [28]. In addition to IN, the capsid protein (CA) has emerged as a second HIV-1 protein that helps to direct PIC targeting to active genes for integration. This review will focus on the role of the CA in HIV-1 integration targeting. Because CA-binding factors prior to integration play important roles in HIV-1 ingress, CA-host interactions pertinent to RTC translocation from the cellular periphery to the nucleus as well as its transport through the cell’s nuclear pore complex (NPC) will be discussed as a lead-in to integration site targeting.

## 2. The Capsid Protein and HIV-1 Core

Appreciation of the role of the CA in HIV-1 ingress and integration targeting is rooted in the understanding of the CA molecule itself as well as its organization within the context of the infectious virus particle. The viral ribonucleoprotein complex is encased in a proteinaceous shell composed of the CA that is oftentimes referred to as the capsid. “Capsid” can accordingly be an ambiguous term with respect to HIV-1 biology because it describes the assembled CA shell in addition to the protein molecule itself. Herein, “CA” refers to the protein molecule. CA molecules come together to form two different higher-order building blocks known as capsomeres (see below). These capsomeres in turn template the formation of the fullerene capsid shell. The shell together with its luminal nucleoprotein components is referred to as the HIV-1 core.

**Figure 2:**
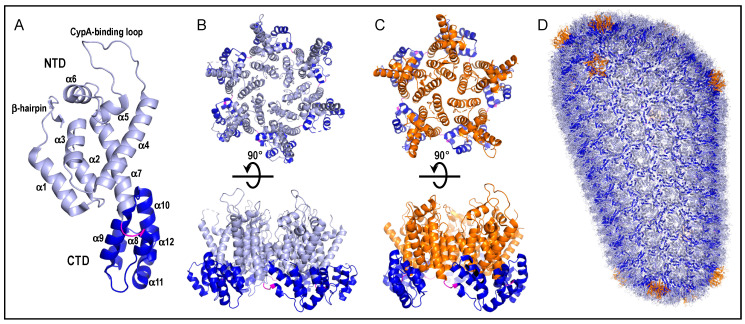
HIV-1 CA, capsomere, and capsid shell structures. (**A**) The structure of the CA monomer from protein data bank (PDB) entrant 4XFY [29] is color-coded to highlight the N-terminal domain (NTD) (residues 1–146; light blue), interdomain linker (residues 147–149; magenta), and C-terminal domain (CTD) (residues 150–231; dark blue). Labels demarcate secondary structural elements. The host factor cyclophilin A (CypA) engages CA via the CypA-binding loop [30]. (**B**) The hexameric capsomere from PDB entrant 4U0D [31]. The upper “top” view represents what would be seen from the outer surface of the core shell. Rotating this view 90° into the plane of the page yields the lower “side” view. Coloring is maintained from panel A. (**C**) The pentameric capsomere (PDB code 3P05) [32]. Upper and lower images are analogous to those in panel B. Orange is used to highlight NTDs of pentameric capsomeres; other coloring is same as panel A. (**D**) Atomic model of assembled core shell (PDB code 3J3Y). The model is composed of 186 hexameric capsomeres and 12 pentamers [33]. Coloring is same as in panels A–C.

CA is composed of two alpha-helical domains, the N-terminal domain (NTD) and C-terminal domain (CTD), which are separated by a short flexible linker (Figure 2A) [34,35]. Intermolecular NTD-NTD and NTD-CTD interactions between adjoining CA molecules stabilize the two different types of ring-like capsomeres. The major HIV-1 capsomere is composed of a hexamer of CA protein while the second capsomere type is a pentamer of CA (Figure 2B,C, respectively) [32,36]. Higher-order CTD–CTD interactions between abutting capsomeres in turn stabilize the honeycomb lattice of the fullerene core shell [37,38]. Twelve pentamers are required to close fullerene-type structures built from repeating hexameric subunits, which are dispersed asymmetrically with 7 at the wide end and 5 at the narrow end to yield the cone shape typical of the HIV-1 core [37] (Figure 2D).

CA is derived from precursor Gag and Gag-Pol polyproteins that are cleaved at specific peptide bonds by the viral protease enzyme during HIV-1 particle maturation (see [39] for review). The immature particle is composed of approximately 2400 Gag molecules [40], from which about half of the liberated CA molecules [41] incorporate into the viral core. Cryo-electron tomography images of HIV-1 cores indicate that they harbor approximately 177 to 209 CA hexamers in addition to the 12 pentamers [33,42] (Figure 2D).

## 3. The Trip to the Nucleus

The capsid shell plays multiple roles during the ingress phase of the HIV-1 replication cycle. Cells are hard wired to respond to viral infection via recognition of foreign nucleic acids (reviewed in [43]). Destabilization of capsid shell integrity via genetic or pharmacological intervention increased cellular innate immune detection of HIV-1, indicating that the shell functions during ingress to shield viral nucleic acids from cellular recognition [44]. Cells have counteractively evolved to recognize and engage unique patterns that are presented via retroviral capsid shells, the consequences of which can restrict viral infection. Tripartite motif (TRIM) 5α forms higher-order oligomers on the outer surface of the capsid shell to induce its premature disassembly, which restricts HIV-1 infection at the reverse transcription step [45,46,47,48]. Binding of myxovirus resistance protein 2 (MxB) to the capsid restricts HIV-1 infection after reverse transcription at the steps of nuclear import and integration [49,50,51,52,53,54,55,56]. The viral capsid additionally serves as a docking platform for cellular dependency factors that aid the transport of the viral core from the cellular periphery to the nuclear membrane as well as its subsequent entry into the nucleus (Figure 1).

The cell cytoplasm is crowded by numerous macromolecular complexes, precluding the inward movement of large particles such as viruses by passive diffusion (reviewed in [57]). Viruses accordingly leverage intracellular trafficking systems to hitchhike to their destinations. The cytoplasmic cytoskeleton is composed of microfilaments, intermediate filaments, and microtubules (ref. [58] for review). Microtubules are polar structures with plus ends located towards the cellular periphery and minus ends embedded within the centrosome or microtubule organizing complex (MTOC), a critical cell division organelle that during interphase often associates with the nuclear membrane (Figure 1). Two different types of motor proteins, dynein and kinesins, associate with microtubules to transport cargoes toward microtubule minus (retrograde) and plus (anterograde) ends, respectively.

A number of cellular factors that participate in microtubule-dependent transport mechanisms have been shown to interact with HIV-1 CA (see [59] and [60] for recent reviews), including microtubule-associated proteins 1 (MAP1) [61], fasciculation and elongation factor zeta 1 (FEZ1) [62,63], diaphanous 2 (Dia2) [64], Bicaudal D2 (BICD2) [65,66], cytoplasmic linker-associated protein 2 (CLASP2) [67], and cytoplasmic linker protein 170 (CLIP170) [68]. Although these studies established contributions from these binding factors to HIV-1 retrograde movement within cells, it is unclear if infection would require the viral core to bind to all of these proteins simultaneously. Because an intact core is composed of approximately 1200 CA molecules, it possesses numerous epitopes for host factor engagement, so binding a wide variety of host factors at once seems plausible. Possibly, factor binding could modulate as transport proceeds, analogous to a relay race whereby a common baton is passed between individuals to complete a single run. Roles of CA binding factors in retrograde HIV-1 transport could also vary depending on the type of infected cell, for example CD4+ T cell versus macrophage.

Simultaneous retrograde and antegrade transport has been proposed to aid uncoating, the process through which capsomeres and CA are shed from the viral core [69]. Though initially thought to occur soon after HIV-1 entry into the cell (see ref. [70] for discussion), advances in imaging technologies have indicated that intact or nearly intact cores are proficient for nuclear translocation, indicating that uncoating may in large part be a nuclear event [71,72,73]. Other CA-binding host factors that have been shown to effect HIV-1 uncoating include cyclophilin A (CypA) [74], TRIM11 [75], transportin 1 (TNPO1) [76], and death domain-associated protein 6 [77].

**Figure 3:**
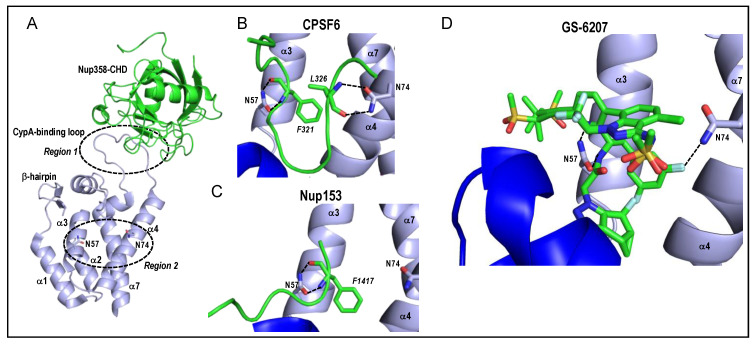
HIV-1 CA and capsomere interaction structures. (**A**) Two common host factor binding regions of CA, which are approximated by dashed circles and labeled Region 1 and Region 2, are superimposed on the structure of the Nup358 cyclophilin-homology domain (CHD) bound to the CA NTD (PDB code 4LQW) [80]. Region 2 is a pocket encompassing alpha helices α3, α4, and α7. Residues Asn57 and Asn74 located on α3 and α4, respectively, are shown as sticks. The orientation and labelling of the CA NTD is preserved from Figure 2A. (**B**) Structure of HIV-1 hexamer capsomere bound by CPSF6 residues 313–327 (green; PDB code 4U0B) [31]. Backbone atoms of CPSF6 residues Phe321 and Leu326, which are shown as sticks with italicized labels, interact with CA residues Asn57 and Asn74, respectively (dashed lines). The CTD visible in the lower left (dark blue) is from a neighboring CA molecule. (**C**) Interaction of Nup153 residues 1407-1423 (green) with HIV-1 CA hexamer (PDB code 4U0D) [31]. The backbone atoms of Phe1417 interact with CA residue Asn57, akin to CPSF6 residue Phe321 (compare with panel B). CA residue Asn74, by contrast, is distal from the Nup153 peptide. (**D**) Interaction of the antiretroviral inhibitor GS-6207 in the Region 2 binding pocket (PDB code 6VKV) [81]. The drug crystallized in two different binding orientations, both of which are shown. Interactions with CA residues Asn57 and Asn74 are highlighted. Stick colorings in panels A–D: red, oxygen; blue, nitrogen; yellow, sulfur; light grey, fluorine.

Two regions of HIV-1 capsomeres, referred to herein as Regions 1 and 2, are common sites of host factor binding. Region 1 encompasses the CypA-binding loop that lies between alpha helices 4 and 5 within the NTD [30] (Figure 2A and Figure 3A). TNPO1 has also been implicated to bind CA via the CypA-binding loop [76]. Unlike the case for CypA, the structure of TNPO1 bound to CA has not been solved experimentally via a wet bench approach such as X-ray crystallography, although a working TNPO1-CA structure was modeled in silico. The second common site for host factor binding on HIV-1 CA (Region 2) is a pocket predominantly formed by alpha helices 3, 4, and 7 of the NTD (Figure 3A) with additional contributions from the CTD of a neighboring CA molecule (Figure 3B,C). It should be noted that the positively-charged central pore of the capsid hexamer composed in part of six apposing Arg18 side chains is an additional common binding pocket for negatively-charged host metabolites including dNTPs [78] and inositol hexakisphosphate or IP6 [79]. Molecular modeling and biochemical experiments indicate that FEZ1 through multiple stretches of poly-Glu amino acid residues also engages the capsomere central pore [63].

## 4. CA Interactions during Nuclear Import

CA is the dominant viral determinant of HIV-1 RTC nuclear import [82] and several CA-binding proteins accordingly play roles in its cytoplasmic-to-nuclear translocation. Such factors can be described as soluble or membrane-bound, with the latter proteins serving as components of the cell’s NPC (Figure 1; also see below). Soluble CA-binding proteins that play roles in HIV-1 RTC nuclear import include CypA [83], TNPO1 [76], BICD2 [65,66], and cleavage and polyadenylation specificity factor 6 (CPSF6) [17,18,71,84].

**Figure 4:**
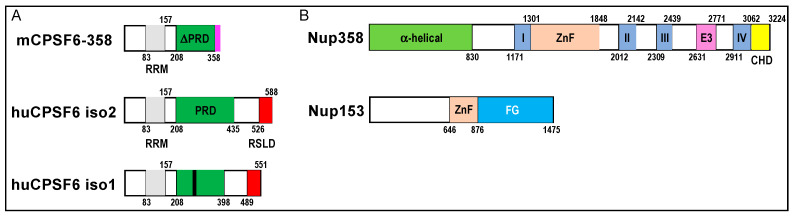
Domain organizations of CPSF6, Nup358, and Nup153. (**A**) Diagram of mCPSF6-358 restriction factor relative to human CPSF6 isoforms 2 and 1; the smaller isoform 1 is the form predominantly expressed in cells. Numbers demarcate domain boundaries. RRM, RNA recognition motif; PRD, proline-rich domain; RSLD, Arg/Ser-like domain. The region of the PRD that confers binding to CA is marked by a black line in human CPSF6 isoform 1. The original mCPSF6-358 construct harbored a heterologous 18-mer sequence (pink) at its C-terminus that did not impact anti-HIV restriction activity [94]. (**B**) Nup358 and Nup153 domain organizations. I–IV, Ran-binding domains I–IV; ZnF, zinc finger domains; E3, E3 ubiquitin ligase domain; CHD, cyclophilin-homology domain; FG, Phe-Gly peptide repeat domain.

CPSF6 is a nuclear protein that as part of the cleavage factor I mammalian (CFIm) complex plays a key role in pre-mRNA 3’ end formation [25,85,86]. CFIm, which is one of many subcomplexes that congregate to form the multicomponent cleavage and polyadenylation (CPA) complex (see [87] for review), is a heterotetramer composed of a dimer of CPSF5 and a dimer of either CPSF6 or CPSF7 [88]. CPSF6, which is predominantly expressed as 551-residue isoform 1 (Figure 4A), is composed of three protein domains. The RNA recognition motif (RRM) domain located in the N-terminal half of the protein mediates the interaction with CPSF5 [89,90]. The proline-rich domain (PRD) in the protein’s midsection mediates binding to HIV-1 CA [91,92] and the C-terminal Arg/Ser-like domain (RSLD) confers binding to the beta-karyopherin transportin 3/SR-2 (TNPO3) [86,93] (Figure 4A). X-ray crystal structures of HIV-1 CA bound by a 15-mer peptide derived from the CPSF6 PRD (residues 313-327; numbering based on isoform 2) revealed that the sidechain of CPSF6 residue Phe321 buried into the Region 2 pocket, with the side chains of CA residues Asn57 and Asn74 contacting the peptide backbone at CPSF6 residues Phe321 and Leu326, respectively (Figure 3B) [31,91]. The RSLD is the functional nuclear localization signal of CPSF6 [86]. Truncation of the mouse CPSF6 isoform analogous to human isoform 2 at residue 358 within the PRD, which additionally removed the RSLD, yielded mCPSF6-358 (Figure 4A). Ectopically expressed mCPSF6-358 localized predominantly to the cell cytoplasm and potently restricted HIV-1 infection at the nuclear import step [94]. CA mutant virus N74D was selected during HIV-1 passage in mCPSF6-358-expressing cells [94] and CA NTD protein carrying the N74D change was accordingly defective for binding to CPSF6_313-327_ peptide in vitro [91]. The N57A change in CA similarly conferred loss of peptide binding in vitro and resistance to HIV-1 restriction in mCPSF6-358-expressing cells [91].

The NPC is composed of approximately 33 nucleoporin (Nup) proteins that arrange in 8-fold rotational symmetry (Figure 1) (see ref. [95] for recent review). The NPC is constructed from several Nup subcomplexes including the coat Nup complex, inner ring Nups, pore membrane Nups, cytoplasmic filament Nups, and nuclear basket Nups. About one-fourth of human Nups harbor multiple Phe-Gly (FG) repeats within otherwise intrinsically disordered regions [96]. The lining of the central pore channel of the NPC with FG Nup proteins confers selective nucleocytoplasmic transport of cargoes whose size is greater than an ~40 kDa globular protein [97].

Recombinant CA or CA-NC protein in the presence of high concentrations of salt (~1 M NaCl) can form higher-order nanotube structures whose surfaces mimic the honeycomb array of oligomeric capsomere hexamers [37,98]. Such reagents are convenient tools for interaction tests with cell factors. Because nanotubes readily sediment via centrifugation, binding factors can be identified in pelleted fractions. Indeed, this assay format initially implicated CPSF6 as a CA-binding factor [94]. Such approaches have since identified Nup proteins Nup358, Nup153, Nup214, Nup88, Nup62, Nup98, Nup107, and Nup153 as CA-interactors [99,100,101,102,103]. Using purified Nup proteins, direct interactions with CA have been demonstrated for Nup358 [80,104] and Nup153 [31,101,105].

The huge Nup358 protein (3224 residues) is a prominent cytoplasmic filament Nup (Figure 1). The C-terminus of Nup358 is a cyclophilin-homology domain (CHD) (Figure 4B) that engages the CypA-binding loop of the NTD in a manner that is highly reminiscent of CypA binding (Figure 3A) [80]. Nup358 plays a role in RTC nuclear import [104,106] although the CHD is notably dispensable for Nup358’s cofactor role in HIV-1 infection [107]. Plausibly, regions of Nup358 upstream from the CHD may also confer binding to HIV-1 CA.

Binding of Nup153 to HIV-1 CA is conferred via its C-terminal FG domain (Figure 4B), with a prominent role for FG repeat 1415-FTFG-1418 [101]. Co-crystallization of a peptide derived from Nup153 residues 1407-1423 with HIV-1 CA hexamers revealed remarkably similar positioning of Nup153 Phe1417 and CPSF6 residue Phe321 in the Region 2 binding pocket, including contacts with CA residue Asn57 (compare Figure 3C with Figure 3B) [31]. Compared to CPSF6, CA residue Asn74 is distal from the bound Nup153 peptide. CA-Nup153 binding during HIV-1 infection was inferred via the restriction activity of an artificial TRIM5-Nup153_896–1475_ fusion construct that harbored the Nup153 FG domain. Consistent with the crystal structures, N57A and N74D CA mutant viruses similarly infected CPSF6-358-expressing cells. While N57A also effectively infected TRIM5-Nup153_896–1475_-expressing cells, N74D infection was restricted by the TRIM5-Nup153_896-1475_ fusion protein [101].

While precise details of HIV-1 RTC nuclear import await clarification, the following scenario can be envisioned. Kinesin-based antegrade transport, perhaps in association with soluble Nup358, shuttles the RTC from the MTOC to the NPC [69] (Figure 1). The Nup358-bound RTC is then shuttled through the NPC, perhaps in conjunction with other FG Nup proteins such as Nup62, Nup98 and/or Nup153, as well as CA-bound TNPO1. As discussed above for retrograde RTC transport through the cell cytoplasm, it is unclear if these interactions would occur simultaneously or sequentially. Upon reaching the nuclear basket, CPSF6 through competing for binding to the common Region 2 pocket liberates the RTC from Nup153’s grasp to enable the RTC’s journey to continue into the nucleus [17,108]. While Nup214 also harbors an FG domain, its role in HIV-1 infection has been mapped to the post-integration egress step of mRNA export from the nucleus to the cytoplasm [99].

## 5. CA Interactions in Integration Targeting

Initial hints of a role for CA in HIV-1 integration site targeting came from studies of chimeric viruses built from parts of HIV-1 and Moloney murine leukemia virus (Mo-MLV), a gammaretrovirus that unlike HIV-1 cannot infect growth-arrested cells [109]. Such fusion viruses initially revealed the role for CA in HIV-1 RTC nuclear import [82]. HIV-1 that carried in place of its own *gag* gene Mo-MLV *gag* determinants that encoded for CA as well as matrix and p12 proteins integrated into regions of chromatin of significantly lower gene-density (~11 genes/Mb) than parental HIV-1 (~20 genes/Mb) [110]. Similar altered integration targeting profiles were observed for wild-type HIV-1 in cells depleted for TNPO3 or Nup358 via small-interfering RNA (siRNA) knockdown [110] and for wild type cells infected with N57A or N74D CA mutant viruses [104]. We subsequently noted that the N74D virus favored gene-sparse regions of chromatin based on the fact that its gene density signature was lower than the value expected by random chance [111]. Using host cell genetics, we subsequently showed that this phenotype was due to loss of CA-CPSF6 binding. HIV-1 integration in HEK293T cells knocked out for *CPSF6*, or U2OS cells depleted for CPSF6 via siRNA, also favored gene-sparse regions of chromatin [25]. While ectopic expression of CPSF6 isoform 1 in *CPSF6* knockout cells in large part restored integration targeting of gene dense regions, expression of CPSF6 mutant F284A, which is defective for CA binding [91,92], did not [25]. Although Jurkat T cells depleted for CPSF6 via transient transfection of Cas9-guide RNA complexes revealed more modest reductions in integration targeting of gene-dense regions (from ~21 to ~15 genes/Mb; random = 7.9 genes/Mb), the N74D mutant virus in large part lost the preference to target gene-dense regions (8.7 genes/Mb) in Jurkat T cells [26].

Our research has recently revealed that speckle-associated domains (SPADs), which are genomic DNA regions in close proximity to nuclear speckles [13,112], track closely with gene-dense regions of chromatin [12]. In silico calculations placed the random chance of SPAD targeting at 2.8%. Remarkably, ~30% to 40% of HIV-1 integrations occurred within SPADs in primary and transformed cell types [12,26]. Specificity of SPAD integration targeting was addressed using the same tools employed for the gene-dense regions targeting studies. For example, N74D mutant virus disfavored SPADs for integration in HEK293T cells [12] and largely avoided them (3.7% SPAD targeting) in Jurkat T cells [26]. While ectopic expression of wild-type CPSF6 restored SPAD integration targeting to *CPSF6* knockout cells, expression of the F284A mutant did not [12]. These data highlighted a critical role for the CA-CPSF6 interaction in SPAD integration targeting [12,26].

Results of HIV-1 imaging experiments have critically informed the role of CA in intranuclear HIV-1 localization and integration site targeting. Viruses unable to engage CPSF6 due to CA changes such as N74D or due to host factor knockout/knockdown uncharacteristically mislocalized to the peripheral region of the nucleus [15,17,18,71,108,113]. Under these conditions, HIV-1 gained significant preference to integrate into LADs [15], heterochromatin domains that physically associate with nuclear lamina (Figure 1) [16] and that are avoided for integration under baseline infection conditions [14,15]. Recent results have highlighted that HIV-1 RTCs/PICs congregate at nuclear speckles in a CPSF6-dependent manner [12,114] for integration into SPADs [12]. Although suggestive that CPSF6 remains bound to CA as the RTC traverses the nucleus, this remains to be determined. CPSF6 could unlatch the RTC from Nup153’s grasp at the NPC basket and then dissociate from the viral complex. Later, perhaps due to the propensity for its RSLD to condense speckle formation [115], CPSF6 could intersect with the RTC/PIC after its arrival at speckles. Because recent data indicates that the HIV-1 core remains largely intact while traversing the nucleus to integration sites [71], we favor a model whereby CPSF6 remains PIC bound until the core uncoats. Additional research is required to discern the fate of PIC-borne CPSF6 after HIV-1 nuclear import.

Other reports have indicated that HIV-1 favors integration into chromatin at the nuclear periphery under basal infection conditions [14,116]. Cross-sample occurrence of genic integration events has identified recurrent integration genes or RIGs, which by definition were genes targeted for integration in minimally two independent studies [14,117]. RIGs can also be defined as genes targeted for HIV-1 integration more frequently than expected based on random chance [15]. Transcriptionally active “hot zones” partition to the nuclear periphery in proximity to NPCs as well as in interior regions proximal to nuclear speckles (Figure 1) [13]. Fluorescence-based imaging of RIGs and proviruses in primary CD4+ T cells indicated preferences of both for the peripheral nuclear region, which suggested a specific architectural arrangement of RIG targeting during HIV-1 integration [14]. In our hands RIGs, like HIV-1 proviruses, were observed more evenly dispersed across nuclear sections of transformed cells and primary T cells, unless, as mentioned, the CPSF6-CA interaction was disrupted [15,118].

RIGs, but not bulk integration sites, were recently determined to track with super-enhancers (SEs) [117], which are regions of the genome enriched for enhancers and epigenetic marks such as H3K4me1 and H3K27ac that are indicative of active chromatin (reviewed in [119]). Because SEs are additionally enriched in SPADs [13], determining whether SEs or SPADs might be an optimal predictor of bulk versus RIG-specific HIV-1 integration targeting is necessarily convoluted. Our recent analyses confirmed that HIV-1 integration overall does not prefer SE regions [12]. Moreover, analysis of a moderate number of RIGs indicated that these preferred genic integration regions tracked more strongly with SPADs than with SEs [12]. Additional work is required to ascertain the contributions of SEs versus SPADs to recurrent targeting of genic integration sites as well as the role of HIV-1 CA in this process.

In addition to Nup358 and CPSF6, CA-binding host factors that have been shown to play roles in HIV-1 integration site targeting include Nup153 [100,111], Nup98 [100], MxB [54], and CypA [104]. Akin to cells depleted for Nup358 or CPSF6, Nup153 depletion reduced integration into gene-dense chromatin regions, although to a comparatively lower extent [111]. Plausibly, RTCs become bottlenecked in the nuclear basket in Nup153-depleted cells, increasing the propensity for the PIC to target nearby LADs for integration, though this has not been formally tested. MxB interacts with various Nups including Nup214 [120] and Nup358 [84]. MxB restriction may retarget PICs from integrating into gene dense regions by inhibiting the Nup358-CA interaction [84]. Interestingly, disruption of the CA-CypA interaction increased HIV-1 integration targeting of gene-dense regions of chromatin [104] and, predictably, SPAD regions. Plausibly, CypA negatively regulates CPSF6’s access to the PIC/RTC, leading to more and/or prolonged CA-CPSF6 binding in the absence of CypA. Because the Nup358 CHD and CypA bind CA Region 1 in highly similar manners, it seems counterintuitive that disrupting the interaction of these two factors with CA results in fundamentally different integration targeting phenotypes. As mentioned earlier, it may not be surprising if regions of Nup358 upstream from the CHD also interact with CA, or alter access of other CA-binding factors, such as CPSF6, to the viral core. Future work that ascertains structures of larger parts of CPSF6, Nup358, and Nup153 bound to CA should inform how these proteins effect each other’s access to the RTC as well as their roles in nuclear import and integration site targeting.

## 6. Conclusions and Perspectives

There are a myriad of CA-binding dependency factors that function during HIV-1 ingress to chaperone the viral core through the cytoplasm, transport the RTC through the NPC, and target the PIC for integration into SPADs. Although it seems likely that the HIV-1 core may stay largely intact during these processes, it at the same time is important to appreciate the plasticity of the core structure. The core, which on average is 60 nm wide at its wide end [42], is seemingly too large to pass through the opening of the average NPC, which is approximately 42 nm [97]. While NPC heterogeneity and dynamics likely contribute to RTC nuclear translocation [73,121], one envisions that an inflexible core would struggle to pass through, regardless of host factor content. At the same time, the HIV-1 capsid shell has evolved to remain operationally intact to shield detection of HIV-1 nucleic acids via the cellular innate immune system [44,122]. Reverse transcription is proposed to effect uncoating [123], and RTCs imaged in vitro show clear signs of partial uncoating [2]. Recent findings for post-nuclear import reverse transcription [72,124] plausibly reconcile incongruities of cytoplasmic uncoating versus shielding of viral nucleic acids from innate immune detection.

One pinnacle of basic science research is informing the development of compounds that can be used to treat human disease. A series of small molecule compounds have been developed that engage Region 2 of the CA. Pioneered by PF-3450074 (PF74), which was a low µM inhibitor of HIV-1 infection [125], next-generation compounds GS-CA1 [126] and GS-6207 (Lenacapavir) [81,127] display exquisite sub-nM antiviral potencies. Structure- and biochemical-based assays indicate that GS-6207 primarily functions to stabilize the HIV-1 capsid shell, which exerts pleiotropic infectivity defects at the ingress steps of RTC nuclear import, integration, and integration site targeting [81]. Due to extensive contacts with Region 2 amino acid residues (Figure 3D) [81,127], GS-6207 effectively inhibited Nup153 and CPSF6 binding to CA nanotubes in vitro [81]. GS-6207 is currently being evaluated in clinical trials as an injectable, long-acting antiretroviral inhibitor of HIV-1 replication [128].

## Data Availability

Not applicable.
